# The Role of Nutraceuticals in Age-Related Ocular Diseases

**DOI:** 10.3390/molecules30173592

**Published:** 2025-09-02

**Authors:** Josè Starvaggi, Carla Di Chio, Fabiola De Luca, Santo Previti, Maria Zappalà, Roberta Ettari

**Affiliations:** Department of Chemical, Biological, Pharmaceutical, and Environmental Sciences, University of Messina, Viale Ferdinando Stagno d’Alcontres 31, 98166 Messina, Italy; jose.starvaggi@studenti.unime.it (J.S.); carla.dichio@unime.it (C.D.C.); fabiola.deluca@unime.it (F.D.L.); spreviti@unime.it (S.P.); mzappala@unime.it (M.Z.)

**Keywords:** natural products, ocular diseases, prevention, phytochemistry, mechanisms of action

## Abstract

Although conventional medicine has seen substantial progress in recent years, there is a growing interest in nutraceuticals, bioactive compounds derived from natural sources such as plants, fruits, and cereals, due to their potential therapeutic applications. These substances have garnered increasing attention for their capacity to support ocular health and to aid in the prevention and management of age-related eye disorders, including age-related macular degeneration (AMD), cataracts, and glaucoma. This review provides a comprehensive and detailed analysis of selected nutraceuticals related to ocular health and diseases. It aims to define their pharmacodynamic properties, to elucidate the molecular and cellular mechanisms underlying their effects and to critically evaluate the current evidence regarding their potential clinical applications. By integrating findings from both preclinical and clinical studies, this review seeks to offer insights into the role of these nutraceuticals in the prevention, management, and adjunctive treatment of various ocular disorders, thereby suggesting future research directions and clinical practice. Notable attention is given to their antioxidant, anti-inflammatory, and neuroprotective properties, which are believed to contribute to the preservation of visual function and the deceleration of disease progression. Elucidating the medicinal benefits of these compounds may open new pathways for complementary or alternative strategies in the prevention and treatment of ocular diseases.

## 1. Introduction

### 1.1. Anatomy of the Eye and Mechanism of Vision

The eye is the sensory organ responsible for vision, capturing light from the external environment and transmitting visual information to the brain. Under physiological conditions, it maintains a spherical shape (anteroposterior diameter, 22–27 mm) and is housed within the bony orbit, where six extraocular muscles enable multidirectional movement [1,2].

Because of constant environmental exposure, the eye requires protection, provided primarily by the tear film, which consists of three layers [3,4,5]. The outer lipid layer, secreted by the Meibomian, Zeis, and Moll glands, reduces evaporation of the aqueous component. The aqueous layer, produced by the lacrimal glands, consists mainly of water, electrolytes, and glycoproteins (e.g., immunoglobulins, lactoferrin, lysozyme) and plays key roles in hydration, cleansing, and defense. The innermost mucous layer, secreted by goblet cells, ensures wettability and protects the ocular surface from pathogens and mechanical friction [1].

Light entering the eye first traverses the tear film and cornea, then passes through the anterior chamber (aqueous humor), iris, pupil, and lens. The anterior segment includes the cornea, which refracts light; the aqueous humor, which maintains intraocular pressure; the sclera, a supportive outer coat; the iris and pupil, which regulate light entry; the crystalline lens, which focuses images; the ciliary body, which adjusts lens accommodation; and the choroid, which supplies nutrients and oxygen [1,2,6].

Posteriorly, light passes through the vitreous humor before reaching the retina. The retina converts light into neural signals via photoreceptor cells—rods (scotopic vision) and cones (color and high-acuity vision). The macula, particularly the fovea, provides sharp central vision, while peripheral regions support broader visual fields. The optic nerve, composed of over one million fibers, transmits these signals to the brain [1,2,6].

Visual perception arises as the brain processes electrical impulses originating from the retina, ultimately generating coherent images [7,8].

### 1.2. Age-Related Eye Diseases

Nowadays, the spread of vision-related disorders has been increasing significantly worldwide, and it is estimated that there are approximately 285 million blind people, of which 65% are visually impaired, with 82% of blindness occurring in individuals over 50 years old [9]. The main age-related eye diseases include cataracts, age-related macular degeneration (AMD), glaucoma, diabetic retinopathy (DR) and dry eye disease (DED) [10,11,12]. Based on studies from the 2000 United States Census report, in 2020, there were 2.95 million people affected by AMD, 3.36 million affected by glaucoma and 30.1 million affected by cataracts [13]. These pathological conditions are often diagnosed at advanced stages, for which treatment is either unavailable or highly problematic [14]. Impaired vision has a profound negative impact on patients’ quality of life, making even simple daily activities—such as walking, reading, writing, and driving—extremely difficult or nearly impossible. Furthermore, it significantly increases the risk of depression and disability, which, especially in old age, lead to an early loss of independence [15,16]. Consequently, research has been intensified, focusing on prevention, and significant investments have in fact been made in these pathologies [17,18]. The predisposing factors associated with risks are innumerable and can be divided into two groups, genetic factors (non-modifiable) and environmental factors (modifiable), as reported in Table 1 [19,20].

The primary genetic factors influencing ocular pathologies are unmodifiable as they are inherent to an individual’s genetic makeup. As previously mentioned, age is the main determinant, with the prevalence of these conditions significantly increasing after the age of 60 [13]. Another important genetic factor is gender difference: although the male–female ratio varies between the various pathologies, women have a greater possibility of becoming blind or visually impaired. In fact, it is estimated that two out of three blind people are women [21]. This may be explained by the greater longevity of the female sex [19]. On the other hand, men have higher mortality rates for all common causes of death: cardiovascular diseases, cancer, infections, accidents and lung diseases [22]. Several explanations for the male–female discrepancy could be attributed to genetic factors, such as the heterogametic sex hypothesis or the length of telomeres or mitochondrial inheritance, or to hormonal factors according to which estrogens should be though to provide greater protection until menopause [23]. It is also known that a light iris color increases the risk factor as it causes the iris to be more susceptible to reactive oxygen species (ROS) attack [20]. On the other hand, environmental factors are associated with lifestyle and are almost all due to damage caused by oxidative stress by ROS; it has been demonstrated that they play a key role in the survival of ocular tissues [24]. Smoking is incredibly harmful because it can reduce the number of antioxidants present in the eye, increases the size and vascularization of the choroid, decreases the optical density of the macular pigment and contributes to the formation of subretinal epithelial deposits [25,26,27]. Another significant environmental factor is excessive body weight (measurable through the BMI: a value higher than 30 represents first-level obesity); in fact, in overweight conditions, the renin–angiotensin system is activated, leading to the production of superoxide, inducing lipid peroxidation and causing a reduction in glutathione peroxidase and erythrocyte glutathione [28]. The following section will explore the primary age-related ocular pathologies in greater detail.

**Table 1:** Genetic vs. environmental factors.

Genetic Factors		Environmental Factors	
Age	[13]	Smoking	[25,27]
Race	[22]	Alcohol consumption	[19,20]
Biological sex	[19,21]	Exposure to sunlight	[13]
Genetic predisposition	[13]	Use of electronic devices: cell phones, tablets, computers	[11]
Ocular pigmentation	[13]	Excessive fat consumption	[17]
		Body Mass Index (BMI)	[28]
		Diabetes	[22]
		Hypertension	[22]
		Cardiovascular diseases	[22]

#### 1.2.1. The Cataract

Currently, this pathology is the leading cause of blindness and visual impairment in developing countries and accounts for 17.7 million cases of blindness worldwide, according to a 2004 study by the World Health Organization [29,30].

Its formation is characterized by a slow, progressive, and irreversible degeneration of the crystalline lens, due in large part to aging [31]. In addition to age-related damage, several other risk factors contribute, including smoking, exposure to UV rays, geographic location, and the use of certain medications (such as steroids), as well as trauma or inflammation of the eye [32,33].

Most of these factors induce oxidative damage, leading to the disruption of cell membrane integrity, the denaturation of lens proteins, DNA degradation, and lens opacification, compromising electrolyte homeostasis [34,35].

Among the ROS, hydrogen peroxide (H_2_O_2_) plays a key role. High concentrations of H_2_O_2_, combined with reduced glutathione levels, have been found in cataract-affected lenses. Glutathione is an important antioxidant that protects the eye by inhibiting the oxidation of sulfhydryl groups in the cell membrane; a decrease in glutathione promotes oxidative stress [36].

There are different types of senile cataracts, categorized by their position within the lens. It is common for individuals to suffer from more than one type simultaneously, as multiple opaque spots can form within the lens [6,37]. The three types are as follows:

(a) Nuclear Cataract: This is the most frequent type, forming in the central portion of the lens. This arises mainly due to the natural aging of the eye.
(b) Cortical Cataract: This is more prevalent in women; it originates in the cortex and may extend to the nucleus. It is very frequently seen in diabetic patients and those with excessive UV exposure.
(c) Posterior Subcapsular Cataract: This develops in the posterior part of the cortex, near the capsule. It often occurs in patients with systemic or ocular diseases and those using certain drugs [6].

Senile cataracts are not the only form; other types may develop earlier in life, including the following:

1. The Congenital Cataract, present from birth;
2. The Juvenile Cataract, appearing at a young age;
3. The Diabetic Cataract, often occurring alongside diabetic retinopathy;
4. The Traumatic Cataract, following severe eye trauma;
5. The Secondary Cataract, which may occur after eye surgeries such as phacoemulsification [38].

Common symptoms of cataracts include blurred vision, photophobia, glare, halos around lights, poor night vision, reduced contrast sensitivity, myopia, astigmatism, and hyperopia [30].

Cataracts are not invariably treated with surgery. In the early or mild stages, visual impairment can often be corrected with updated eyeglass prescriptions or other conservative measures. When the condition progresses and vision loss becomes more significant, phacoemulsification is the most employed surgical technique. This ultrasound-based procedure uses a fine probe inserted through a 2.8–3.2 mm corneal incision to fragment the opacified crystalline lens, which is then removed. Subsequently, an artificial intraocular lens is implanted through the same incision to restore visual function. Presbyopia often follows the procedure. Other side effects, though rare, may include refractive errors, capsular opacification, cystoid macular edema, retinal detachment, infections, and suprachoroidal bleeding—which, if severe, may lead to vision loss [39].

#### 1.2.2. Glaucoma

Glaucoma is a degenerative neuropathy affecting the retinal ganglion cells. It is currently recognized as the second leading cause of blindness worldwide and the third leading cause of visual impairment. The degeneration of these cells causes progressive optic atrophy that can lead to a complete loss of vision [40,41].

Globally, approximately 3.54% of people aged 40 to 80 are affected by glaucoma, totaling about 64.3 million people, with projections reaching 111.8 million by 2040 [42]. Women are more frequently affected, accounting for 59.1% of total cases, likely due to their longer life expectancy [41].

A characteristic of this pathology is elevated intraocular pressure, which—depending on the condition of the sclero-corneal trabecular meshwork—allows glaucoma to be classified into two types [43]:

(a) Open-angle (chronic) glaucoma is the most common form (approximately 80%). It is characterized by difficulty in the outflow of aqueous humor through the sclero-corneal trabecular meshwork. It progresses slowly and is primarily treated with β-blockers.
(b) Closed-angle (acute) glaucoma is more aggressive, caused by a physical obstruction of the sclero-corneal trabecular meshwork, which prevents the outflow of aqueous humor. It progresses rapidly and can lead to blindness within days [36,41].

Regardless of the type, glaucoma results in the loss of retinal ganglion cell axons, leading to the thinning of the neuroretinal rim and damage to the optic nerve. This can be accompanied by retinal ischemia due to the overstimulation of ionotropic glutamate receptors, the excessive activation of which may cause excitotoxic cell death [41].

Diagnosing glaucoma is challenging due to its often asymptomatic progression until advanced stages [44]. However, several factors can aid in early identification and prevention. Risk factors include smoking, diabetes, hypertension, and genetic predisposition. Additional risk factors include high myopia, optic nerve structure, vascular anomalies, and abnormalities in ocular perfusion pressure [45,46,47].

Therefore, effective diagnosis is only possible following a comprehensive eye examination, including intraocular pressure measurement and visual field testing—such as SAP (Standard Automated Perimetry), FDT (Frequency Doubling Technology), or SWAP (Short-Wavelength Automated Perimetry) [46,48].

At present, no definitive cure exists for glaucoma; however, disease progression can be prevented or delayed through a reduction in intraocular pressure. Such elevation may arise either from increased aqueous humor production or from impaired outflow. Current therapeutic strategies include pharmacological treatment (e.g., β-blockers), laser procedures, and surgical intervention [49]. Increasing attention has recently been directed toward nutraceuticals, which are emerging as promising adjunctive approaches owing to their anti-inflammatory and antioxidant properties [36,50].

#### 1.2.3. Age-Related Macular Degeneration (AMD)

In highly industrialized countries such as Western Europe, North America, Australia, and the Asia–Pacific region, AMD is one of the leading causes of blindness in patients over 60 years of age, affecting approximately 5% of the world population [49,51,52]. Estimates suggest that AMD, together with glaucoma and DR, will become even more common in the coming years due to increased life expectancy and greater accessibility to cataract surgery [53].

The retina’s susceptibility to developing AMD is not limited to advancing age or oxidative stress alone; in fact, several impactful risk factors contribute to its onset, such as the following:

(a) Geographical Area and Ethnicity: In the United States, individuals of white ethnicities account for 54.4% of cases of blindness due to maculopathy [13].
(b) Genetic Profile and Family History: A family history of AMD significantly increases the risk of developing the disease.
(c) Cardiovascular Diseases, Arterial Hypertension, Obesity, and Diabetes.
(d) Hyperopia and Lens Opacity.
(e) A Diet Rich in Saturated Fats and Poor in Essential Omega-3 Fatty Acids [54,55]. This pathology can initially develop asymptomatically before progressing into one of two types:

Dry Maculopathy (Dry or Atrophic AMD): This variant, which accounts for approximately 80% of cases, is generally less severe than the wet type. It is defined by the buildup of lipids and cellular debris that give rise to deposits beneath the retina, known as drusen. These deposits compromise regions essential for vascular supply, thereby impairing the nourishment of macular cells and ultimately leading to their gradual degeneration. This explains why a high intake of saturated fats is a significant risk factor and highlights the importance of omega-3 integration (anticholesterolemic agents) in preventing this condition. Symptoms typically involve visual impairment and, in rare cases, atrophied retinal areas. Both eyes are usually affected simultaneously. In addition to dietary management, this form can also be treated with laser surgery [56].

Wet Maculopathy (Wet or Exudative AMD): This is the most aggressive form and the leading cause of AMD-related blindness. It may evolve from the dry form. In response to insufficient nutrients and oxygen, the macular cells release VEGF (*Vascular Endothelial Growth Factor*), which promotes angiogenesis. However, the newly formed blood vessels are fragile, allowing the leakage of fluids and blood, leading to scar tissue formation in the sub-macular area. Symptoms include initial difficulty in focusing images followed by a rapid loss of central vision (while peripheral vision remains intact). Although this form typically starts in one eye, the risk of it developing in the other eye is high. It is commonly treated with monoclonal antibodies—anti-VEGF drugs such as Bevacizumab and Ranibizumab—that inhibit abnormal blood vessel growth [56,57].

The diagnosis of AMD is performed using ophthalmoscopy, typically via Optical Coherence Tomography, which allows for highly precise scanning of the cornea, iris, and especially the retina to detect any damage [56].

Given the global impact of AMD, several research studies—known as AREDS—have been conducted to explore appropriate nutritional interventions. Two studies were carried out, with the second (Table 2) designed to address and improve upon the limitations identified in the first (Table 3).

Vitamin C and Vitamin E: These are used as primary natural antioxidants to combat the formation of free radicals in the retina.

β-Carotene (later replaced with Lutein and Zeaxanthin): This is initially used as an antioxidant; β-carotene had to be replaced in patients who were smokers or ex-smokers, as it significantly increased the risk of lung cancer.

Zinc serves as a crucial cofactor for several ocular enzymes owing to its function as a reducing metal. Nevertheless, the high dosage administered in the initial AREDS-1 trial (80 mg) was associated with recurrent hospitalizations for gastrointestinal and genitourinary complications. Consequently, in the AREDS-2 trial (Table 3), the zinc dose was reduced to 25 mg. Regarding omega-3 fatty acids (ω3), their inclusion in AREDS-2 (350 mg DHA and 650 mg EPA) was based on the rationale that drusen formation results from the subretinal accumulation of lipids and cellular debris, contributing to AMD. Since omega-3 fatty acids primarily act by lowering triglyceride levels, their supplementation was hypothesized to mitigate drusen development. However, findings from AREDS-2 indicated that DHA and EPA supplementation did not consistently lead to a reduction in drusen burden [56].

#### 1.2.4. Diabetic Retinopathy

DR accounts for 1% of all visual impairments and 1% of blindness caused by ocular pathologies. It is among the leading causes of total blindness and global visual impairment [49,51]. Furthermore, it represents the most common complication of diabetes mellitus (of any type), affecting approximately two-thirds of patients. Other conditions that may arise concurrently from diabetes include peripheral neuropathy, peripheral arterial disease, diabetic nephropathy, diabetic foot, heart attack, and stroke, as well as the previously mentioned AMD, glaucoma, and cataract [58].

DR is a progressive microvascular disorder that develops in two phases, often accompanied by diabetic macular edema (DME) [59]. It is classified as follows:

Non-Proliferative Diabetic Retinopathy (NPDR or Early Diabetic Retinopathy): This is the initial phase of the disease and is characterized by increased capillary permeability. In this phase, as in wet AMD, blood vessels tear, causing fluids and blood to leak, leading to hemorrhages and edemas (including DME). Over time, it may progress to the proliferative form.

Proliferative Diabetic Retinopathy (PDR): This stage represents the advanced phase of the disease and is characterized by a marked upregulation of VEGF expression. The incidence of edema and hemorrhages increases, culminating in macular ischemia, vitreous hemorrhage, and, in some cases, retinal detachment [59,60]. In the early stages, the disease is asymptomatic and typically diagnosed through OCT. Symptoms appear as the disease progresses and may include the following: myodesopsia (floaters), blurred vision, poor night vision, dark spots in the visual field, faded colors, and reduced focus [61]. The primary treatment for DME is intravitreal anti-VEGF therapy, while photocoagulation is the preferred treatment for PDR [62].

## 2. Mechanism of Free Radicals and Endogenous Antioxidants

Oxygen (O_2_) is one of the essential elements for the survival of all living beings, as it is necessary for cellular (aerobic) respiration, during which it undergoes oxidation–reduction reactions. However, some of these reductions are incomplete, and part of the oxygen forms highly reactive and unstable species known as ROS [63].

The main ROS formed within the human body are as follows:

Superoxide Anion (O_2_^−^): The most abundant ROS and the first to form. Due to its very short half-life (a few milliseconds), it is unable to directly attack biological macromolecules. However, it can initiate chain reactions that lead to the formation of other radicals. Additionally, it is partially used by the body to help eliminate certain pathogenic microorganisms [64].

Hydrogen Peroxide (H_2_O_2_): A small, non-radical molecule capable of easily crossing biological membranes. It can generate other ROS and acts either as an oxidizing agent (in acidic environments) or a reducing agent (in basic environments) due to the oxidation state of oxygen being +1 [65].

Hydroxyl Radical (OH): Despite its extremely short half-life, it is highly reactive and capable of damaging any type of macromolecule. It cannot be eliminated by enzymatic reactions, making it the most powerful and harmful ROS to the human body [66].

As a result, oxidation caused by these species primarily affects biological macromolecules by altering amino acids, modifying membrane lipids (compromising their structure), or damaging DNA by fragmenting nucleic acids [67].

Since ROS production is a physiological process, the body has developed a set of defense mechanisms known as endogenous antioxidants or scavengers to maintain a balance between their production and elimination. A disruption in this balance leads to a condition known as oxidative stress [64].

The main scavenger enzymes include the following: 

Superoxide Dismutase (SOD): This is an enzyme that catalyzes the neutralization of superoxide anions by converting them into hydrogen peroxide and oxygen. In the human body, SOD exists in three forms: SOD1, found in the cytoplasm, containing copper and zinc; SOD2, located in the mitochondria, also containing copper and zinc; SOD3, found extracellularly and containing manganese [64].

$$2\;{O_{2}}^{-}\;+2H^{+}⇌\;O_{2}\;+H_{2}O_{2}$$

Catalase (CAT): This is an enzyme (specifically a hemoprotein) capable of detoxifying the body from hydrogen peroxide by generating oxygen and water. This reaction occurs thanks to the four ferrous groups contained within the protein [68].

$$2\;H_{2}O_{2}⇌\;O_{2}\;+2\;H_{2}O$$

Glutathione Peroxidase (GSH-Px): Another enzyme that catalyzes the transformation of hydrogen peroxide. There are several isoforms (currently eight identified) present almost ubiquitously within the organism. They contain selenium [68].

$$2\;Glutathione+H_{2}O_{2}\;⇌Glutathione\;Disulfide+2\;H_{2}O\;$$

The imbalance caused by oxidative stress leads to excessive production of free radicals, increasing the likelihood of developing various pathologies throughout the body. The most frequent include cancer, neurodegenerative diseases, heart disease, asthma, aging, infertility, dermatitis, hypertension, rheumatoid arthritis, and diabetes [69].

The eye is especially susceptible to damage by ROS due to continuous exposure to sunlight, which generates photooxidation in the retina. Additional contributing factors include the high content of mitochondria, which require a large amount of oxygen and thus increase the risk of free radical accumulation, as well as the massive presence of polyunsaturated fatty acids (PUFAs) in the retina, which are particularly sensitive to lipid peroxidation [70].

As a result, major ocular pathologies may develop, including AMD, DR, cataracts, dry eye disease (DED), uveitis, retinitis pigmentosa, toxic optic neuropathies, and other ocular surface disorders [71,72].

In addition to the factors already mentioned, other causes of radical formation include smoking, constant use of electronic devices, an unbalanced diet rich in fats, atmospheric pollution, and alcohol consumption [20,26].

New therapeutic approaches focus on natural antioxidant components derived from specific foods and nutrients to reduce the recurrent use of pharmaceuticals and promote the use of natural extracts [73].

## 3. Definition of Nutraceuticals, Functional Foods, Supplements, and Nutraceuticals

Nutraceuticals are bioactive compounds derived from foods or food components that confer health benefits beyond basic nutrition, including the prevention and management of ocular diseases. Nutrition plays a pivotal role in human health, and consequently, various scientific disciplines—such as nutraceutics and nutrigenomics—together with organizations like the European Food Safety Authority (EFSA) have established guidelines to promote a healthy diet. Over the past five decades, functional foods, dietary supplements, and nutraceuticals have been introduced as nutritional adjuvants to address dietary deficiencies. Functional foods are defined as foods which, in addition to their intrinsic nutritional value, can enhance normal physiological functions and, in certain cases, contribute to the prevention or even treatment of disease. This term was coined in the 1980s in Japan, where criteria were established to define what were called FOSHU (Foods for Specified Health Use). Their main characteristics are as follows.

They must not be presented as formulations (e.g., capsules, tablets, or powders), they must be part of the daily diet in their natural form (i.e., not fortified foods), and they must have a functional impact on human physiology [74,75].

Functional foods can be considered phyto-complexes, as they contain a variety of molecules with therapeutic potential. However, studies have shown that, compared to their individual components, they often demonstrate lower efficacy in affecting human physiology [74]. Nonetheless, their consumption is strongly recommended, as this category includes all types of traditional fruits and vegetables that support the prevention and treatment of many pathologies, especially ocular ones.

It has been observed that consumption of green cabbage, kale, carrots, and peaches is associated with a reduced incidence of glaucoma—from 69% to 47% [76].

A major concern with these products is the absence of adequate regulation, which can compromise their quality, dosage accuracy, and overall safety, while also increasing the risk of adverse reactions such as allergies [77]. For instance, functional foods enriched with honey or salicylates may lead to gastrointestinal disturbances, nausea, or vomiting when consumed in excessive amounts. Likewise, certain plant-based foods, such as St. John’s wort, can interfere with drug pharmacodynamics by reducing their bioavailability [78]. In contrast to functional foods, food supplements are deliberately formulated dietary products that are commercially available in various forms, including capsules, tablets, granules, and gels [75]. Their broad accessibility reflects their expanding role as adjuvant interventions across multiple areas of health. Commonly marketed examples include sports supplements (e.g., carnitine and BCAAs), multivitamin–multimineral combinations (e.g., calcium, magnesium, and potassium), and polyunsaturated fatty acid (PUFA) supplements such as omega-3 [75,79].

Within the broader category of dietary supplements, nutraceuticals are particularly noteworthy for their wide-ranging health applications. Although Italian law classifies them as a subset of supplements, their functions differ substantially. Whereas supplements are primarily intended to correct nutritional deficiencies by supplying concentrated nutrients, nutraceuticals are designed to exert preventive and supportive effects in the management of specific pathologies—without necessarily providing additional nutrients [75,80]. Moreover, unlike functional foods, nutraceuticals are produced under rigorous quality controls, allowing precise identification of the name and quantity of ingredients. This significantly reduces the risk of adverse effects [80].

Nutraceuticals can be classified based on their chemical nature (lipid, protein, carbohydrate, micronutrient, or microbial), their mechanism of action (physiological or metabolic), or their origin (vegetable, animal, or microbial) [81]. They are further divided into two main categories: traditional and non-traditional [82].

### 3.1. Non-Traditional Nutraceuticals

Non-traditional nutraceuticals, also referred to as artificial or synthetic, are produced using biotechnology. This category includes two main groups: recombinant nutraceuticals and foods fortified with nutraceuticals [82,83].

#### 3.1.1. Recombinant Nutraceuticals

Recombinant nutraceuticals include all food products that provide energy and are produced through various biotechnological methods and genetic engineering techniques [83]. An interesting example is a study by Malbaša et al., which utilized kombucha, a carbonated beverage obtained through the fermentation of sweetened tea—containing various yeast strains such as *Candida* sp. (Berkhout, 1923), *Saccharomyces cerevisiae* (Hansen, 1883), *Zygosaccharomyces* sp. (Barnett, 1983), *Saccharomyces bisporus*, and *Saccharomycodes ludwigii* (Hansen, 1904)—cultivated on black or green tea substrates. The antioxidant properties of kombucha were confirmed by its ability to neutralize hydroxyl radicals and 2,2-diphenyl-1-picrylhydrazyl (DPPH). These beneficial effects are primarily attributed to the presence of polyphenols, vitamin C, vitamin B2, and citric acid in the beverage [84].

Another example of a recombinant nutraceutical is lysozyme (RhLYZ), also known as muramidase, which is well known for its bactericidal properties. It is a protease synthesized by white blood cells and plays a key role in the body’s defense system due to its proteolytic activity, which enables it to break down bacterial cell walls. Yang et al. demonstrated its nutritional and immunological benefits when extracted from transgenic cattle, highlighting the significant medical advancements made possible through biotechnology [85].

While the direct role of rhLYZ in cataract prevention remains under investigation, its presence in tear fluid suggests a potential protective effect against oxidative stress and inflammation, factors implicated in cataractogenesis. A study developed lysozyme-triggered nanodiamond contact lenses for glaucoma treatment, indicating the potential of lysozyme in ocular applications. RhLYZ has been utilized in drug delivery systems to enhance the efficacy of glaucoma treatments. A study demonstrated that lysozyme-triggered nanodiamond contact lenses can improve the sustained release of anti-glaucoma drugs, potentially enhancing therapeutic outcomes. Romanovskaya et al. developed lysozyme-containing eye drops with enhanced stability and activity under inflammatory conditions, suggesting potential applications in treating dry eye symptoms associated with AMD [86,87].

#### 3.1.2. Foods Fortified with Nutraceuticals

Foods fortified with nutraceuticals are products to which nutrients have been added beyond their original composition [83]. Recently, they have become the focus of research aimed at improving the health of individuals with specific nutritional deficiencies, which may increase vulnerability to various diseases [80]. A review conducted by Cormick et al. highlighted that calcium-fortified products, particularly milk, led to increased calcium intake, greater bone mineral density in the hip and femoral neck, and increased height in children [88]. Other studies have reported positive effects on bone mass in individuals at risk of fractures following the use of such fortified formulations [89]. Similarly, flour fortified with folic acid has been shown to effectively prevent neural tube defects such as encephalocele and spina bifida [90,91]. Finally, iodized salt has proven effective in preventing the development of goiter and non-immune hypothyroidism, both of which can lead to mental impairment [92].

### 3.2. Traditional Nutraceuticals

Traditional nutraceuticals, also known as natural nutraceuticals, are derived from microbial, animal, plant, or mineral sources. They are divided into three main groups, chemical components, enzymes, and probiotics, each of which includes further subgroups. Among the chemical components, subcategories such as phytochemicals, nutrients, and herbal extracts are distinguished [82,83].

#### 3.2.1. Enzymes

Enzymes are proteins composed of 100 to 2000 amino acid residues, forming polypeptide chains that create an active site where substrates bind. The conformational change in this site is responsible for the specific catalytic activity toward a single substrate [93]. It has been shown that the main role of these enzymes is to enhance the effectiveness of other nutraceuticals and supplements [83,94]. For example, pectinases and cellulases obtained from an extract of *Ecklonia* (Sonder, 1845) (an alga belonging to the Lessoniaceae family) have been shown to influence obesity in murine models by reducing glutamate-pyruvic transaminase, insulin, and leptin levels [95]. Additionally, soy protein hydrolysates combined with proteases such as papain and trypsin have been shown to induce an immunomodulatory effect by increasing the production of lymphocytes and phagocytic activity in in vitro models [96].

#### 3.2.2. Probiotics, Prebiotics and Symbiotics

The presence of probiotics—vital microorganisms that, when administered in adequate quantities, provide health benefits to the host—has been widely studied. The most commonly available species include *Saccharomyces boulardii* (Boulard, 1923), *Bifidobacterium* (Orla-Jensen, 1924), and *Lactobacillus* (Beijerinck, 1901) [83,97]. In a study by Nagashima et al., it was demonstrated that a preparation containing *Enterococcus faecium* WB2000 (Orla-Jensen, 1919), *Lactobacillus pentosus* TJ515 (Mees, 1934), and resveratrol increased the elasticity of the crystalline lens in murine models. This effect was observed both with long-term administration (40 weeks of 0.042, 0.007, and 0.088 mg/day, respectively) and short-term administration (4 weeks of 0.21, 0.007, and 0.44 mg/day, respectively) [98].

*Saccharomyces boulardii* has demonstrated antioxidant activity and a notable ability to attenuate various gastrointestinal diseases, such as pseudomembranous colitis, as well as to reduce adverse effects caused by *Helicobacter pylori* (Warren, 1984) infection. It has been shown to neutralize members of the Enterobacteriaceae family, such as *Escherichia coli* (Migula, 1895), *Salmonella* (Lignières, 1900), and *Vibrio cholerae* (Pacini, 1854). In addition, it increases the expression of anti-inflammatory interleukins, such as IL-1, IL-5, IL-10, and IL-12, while decreasing the expression of pro-inflammatory interleukins, such as IL-6 and TNF-α [99,100].

*Bifidobacteria* and *Lactobacilli* have also been shown, with the intake of one capsule per day (69 g), to play a key role in restoring obesity-induced dysbiosis [101].

Prebiotics are organic, non-digestible substances that confer health benefits by modulating the growth and activity of probiotics. They pass through the digestive tract largely intact and serve as a fermentable nutritional substrate for the intestinal microflora, producing various beneficial effects on human health. The most common prebiotics are oligosaccharides, in particular inulin (a soluble fiber from the Asteraceae family, consisting of long chains of fructose) and FOS (fructo-oligosaccharides, present in various fruits, vegetables, and plants). Other oligosaccharides include TOS (trans-galacto-oligosaccharides), GOS (gluco-oligosaccharides), and SOS (soy-oligosaccharides). Prebiotics also perform numerous functions within the human body, such as decreasing fecal pH, increasing mineral bioavailability, and exerting a hypocholesterolemic effect [101].

Finally, symbiotics are products that simultaneously contain both probiotics and prebiotics [101]. Symbiotics, combining probiotics and prebiotics, may help restore gut microbiota balance, reduce systemic inflammation, and enhance antioxidant defenses, potentially slowing cataract progression [102].

Emerging evidence suggests that gut microbiota composition influences systemic inflammation and immune responses, which may impact AMD progression. Modulating gut microbiota through symbiotics could be a potential therapeutic strategy for AMD [103]. Recent studies have identified alterations in gut microbiota composition in glaucoma patients, indicating a potential link between gut health and glaucoma pathogenesis. Symbiotics may help modulate gut microbiota, reduce inflammation, and protect retinal ganglion cells, offering a novel approach to glaucoma management [104].

#### 3.2.3. Nutrients

Nutrients are substances derived from both animal and plant sources, including peptides, carbohydrates, fatty acids, vitamins, and minerals. They play a key role in the development of numerous pathologies, such as tumors, cardiovascular diseases, degenerative diseases, ocular diseases, and inflammatory conditions [83,95].

##### Bioactive Peptides

**Figure 1:**
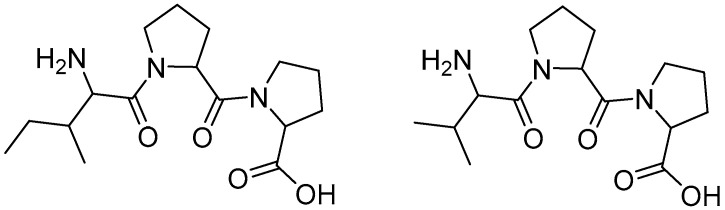
Structure of lactotripeptides Ile-Pro-Pro and Val-Pro-Pro.

Bioactive peptides are compounds composed of amino acids linked by peptide bonds. They are typically released through the action of proteases of enzymatic or microbial origin, which break down the proteins present in food. These peptides are defined as protein fragments that exert a beneficial effect on the human body. The typical sources of bioactive peptides are of animal origin (such as meat, eggs, and milk), although new alternative plant-based sources—such as soy, oats, and wheat—are increasingly being explored [105]. Bioactive peptides are mainly classified according to their mechanism of action, which includes immunomodulatory, antihypertensive, antimicrobial, opioid, antioxidant, anti-inflammatory, and antithrombotic activities [106]. Interestingly, these molecules have attracted attention in nutraceutical research due to their antihypertensive properties, which are achieved through the inhibition of angiotensin-converting enzyme (ACE). Among the best-known examples are the lactotripeptides isoleucine-proline-proline (Ile-Pro-Pro) (Figure 1) and valine-proline-proline (Val-Pro-Pro) (Figure 1), the efficacy of which appears to be linked to the presence of proline residues. Furthermore, caseino macropeptide, obtained from milk, has demonstrated significant antithrombotic effects [107].

Another important example is azurocidin (CAP37), which is obtained from neutrophil granulocytes and is particularly effective against bacteria involved in corneal infections, such as *Pseudomonas aeruginosa* (Schroeter, 1872) and *Staphylococcus aureus* (Rosenbach, 1884). Its notable antimicrobial effects, studied in mice, appear to be mediated by the activation of the protein kinase C (PKC) signaling pathway in corneal epithelial cells. This mechanism promotes the migration of these cells and accelerates wound closure following the topical administration of CAP37 at concentrations of 250–500 ng/mL. Furthermore, intrastromal injection has been shown to increase the expression of IL-7, IL-15, and IFN-γ, thereby enhancing corneal epithelial recovery [108,109,110].

##### Bioactive Carbohydrates

Bioactive carbohydrates are primarily obtained from plants such as algae, wood, dietary fibers, or herbs; from animal tissues such as hyaluronic acid, chondroitin sulfate, or heparin; and from microorganisms. Chemically, polysaccharides may have a linear structure, known as homoglycans (e.g., cellulose), composed of repeated units of the same monosaccharide, or a branched structure, referred to as heteroglycans (e.g., heparin), which consist of different types of monosaccharides. These polysaccharides exhibit a wide range of biological activities, including antioxidant, antimicrobial, antithrombotic, hypoglycemic, and antitumor properties [111,112].

A noteworthy example is the class of sulfated polysaccharides derived from seaweed, mainly alginate, laminarin, and fucoidan. These compounds are capable of inhibiting lipid peroxidation, neutralizing free radical species such as nitric oxide, superoxide anion, and hydroxyl radical, and inducing the activity of glutathione peroxidase and superoxide dismutase [113]. Among them, fucoidan stands out due to its potent antithrombotic and anticoagulant properties, achieved by inhibiting factor Xa, thrombin, and both the intrinsic and extrinsic coagulation cascades [114]. Furthermore, the administration of 400 mL/day has been explored in oncological therapy as an anti-inflammatory agent, reducing pro-inflammatory cytokines such as IL-6, TNF-α, and IL-1β [115].

##### Fatty Acids

Fatty acids consist of a long non-polar hydrocarbon chain ending with a carboxylic acid group. They are classified as follows: saturated fatty acids, which contain only single bonds in the hydrocarbon chain, and unsaturated fatty acids, which contain one (monounsaturated) or multiple (polyunsaturated) double bonds [112]. Of particular nutraceutical interest are PUFAs, due to their significant health benefits and important roles in human physiology.

Polyunsaturated Fatty Acids

**Figure 2:**
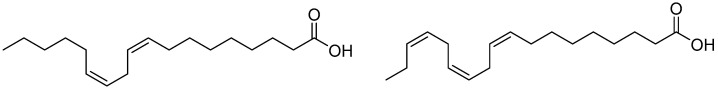
Structure of polyunsaturated fatty acids LA and ALA.

Among the PUFAs, the most important are the essential fatty acids (EFAs). These are vital for the human body, which is unable to synthesize them and must therefore obtain them through the diet. They include the following:

- Linoleic acid (LA), formula C18:2, the precursor of Omega-6 (ω-6) (Figure 2);
- α-linolenic acid (ALA), formula C18:3, the precursor of Omega-3 (ω-3) (Figure 2).

Once ingested, essential fatty acids are converted into semi-essential fatty acids. Linoleic acid (LA) gives rise to arachidonic acid (AA) and type 2 prostaglandins (PGE2), both of which are pro-inflammatory, while alpha-linolenic acid (ALA) leads to the formation of eicosapentaenoic acid (EPA) and docosahexaenoic acid (DHA), which are anti-inflammatory [110].

Omega-3 fatty acids are of great nutritional importance due to their effective anti-inflammatory action, which competitively inhibits AA. For this reason, it is advisable to maintain an optimal ω-6/ω-3 ratio of 4:1. Nowadays, in Western countries, this ratio ranges from 10:1 to 20:1, where a excess of Omega-6 is associated with increased synthesis of thromboxane A2, leukotriene B4, interleukin-1β (IL-1β), interleukin-6 (IL-6), and tumor necrosis factor (TNF-α). This imbalance contributes to the development of chronic inflammatory diseases such as cardiovascular diseases, cancer, obesity, and autoimmune disorders [116,117].

The main dietary sources of ALA are plant-based products such as flaxseeds and whole grains, while EPA and DHA are found directly—without the need for synthesis from ALA—in oily fish [116].

**Figure 3:**
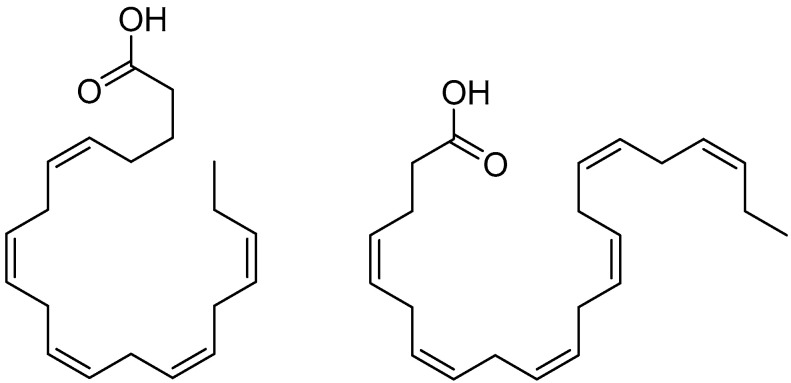
Structure of polyunsaturated fatty acids EPA and DHA.

Eicosapentaenoic acid (EPA) (Figure 3) and docosahexaenoic acid (DHA) (Figure 3) appear to play crucial roles in several neurovascular ocular diseases, including DR, AMD, and retinopathy of prematurity, conditions for which current treatments often cause significant side effects.

However, clinical and experimental evidence suggests that enhanced supplementation with these ω-3 fatty acids may alleviate adverse effects [118]. Despite conflicting results between basic and clinical research, these PUFAs play a crucial role in the pathogenesis of glaucoma by influencing both the reduction in intraocular pressure (IOP) and the survival of retinal ganglion cells (RGCs). Endogenous prostaglandins (PGs) derived from PUFA metabolism, through the activation of EP4 and FP receptors, reduce IOP values. At the same time, they reduce inflammation and oxidative stress, which are largely responsible for RGC dysfunction or death [119,120].

A valuable example is patients affected by pseudo-exfoliative glaucoma (PEX), who benefited from the intake of a DHA-rich formulation due to reductions in oxidative stress and inflammation. The efficacy of PUFAs in glaucoma treatment also appears to be linked to their ability to improve endothelial function and counteract atherosclerosis [121]. A controlled study in male Wistar rats demonstrated that the antioxidant and anti-inflammatory effects of lutein are enhanced in the presence of EPA and DHA [122]. Evidence further indicates that a micellar formulation of lutein combined with EPA and DHA exerts a modulatory effect on crystalline chaperonins [123]. In addition, Chang et al. reported a significant reduction in free DHA fatty acids in patients with senile cataracts [124]. Research into the molecular mechanisms of retinal aging has identified a key role for the enzyme ELOVL2 (elongation of very-long-chain fatty acids-like 2), which catalyzes the elongation of ω-3 and ω-6 fatty acids, and has been directly associated with the development of age-related macular degeneration (AMD). Indeed, reduced levels of these polyunsaturated fatty acids have been detected in the ocular tissue of AMD patients, potentially implicating ELOVL2 in disease pathogenesis. Moreover, DHA appears to confer protective effects by stimulating endogenous antioxidant production and promoting the selective autophagy of misfolded proteins, thereby mitigating disease onset [125].

Furthermore, in subjects affected by DR, levels of very long-chain PUFAs (VLC-PUFAs) were found to be lower than in healthy subjects of the same age. For this reason, Gorusupudi et al. concluded that dietary enrichment with ω-3 fatty acids contributes to reducing the risk of diabetes and DR [126].

Finally, several studies suggest that specialized pro-resolving mediators (SPMs), derived from PUFAs, can support ocular surface health and immune homeostasis, thanks to the high expression of SPM pathways and receptors on the ocular surface [127].

##### Vitamins

Vitamins are a class of compounds belonging to the category of micronutrients, necessary to satisfy various physiological needs.

Vitamin A

**Figure 4:**
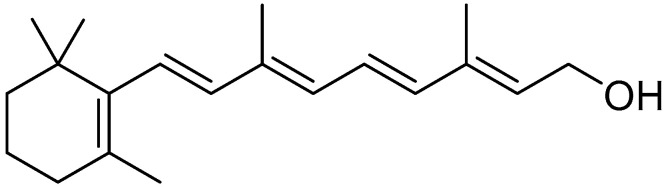
Structure of retinol.

Among the fat-soluble vitamins, vitamin A (Figure 4) stands out for its fundamental role. It is mainly represented by retinol (vitamin A1) and its retinoid analogs, such as retinaldehyde and retinoic acid.

These compounds are essential for countless vital functions, such as the regulation of cellular differentiation, gene expression, embryonic development, and the immune system. Additionally, they play a key role in vision by improving night vision and promoting the development of the cornea and conjunctiva [128].

**Figure 5:**
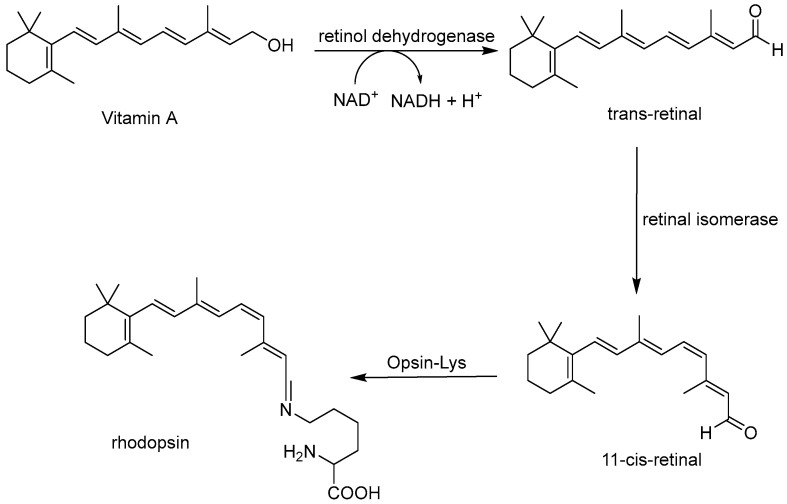
Synthesis of rhodopsin.

Vitamin A can be obtained both in the form of retinol from animal-based foods (such as eggs, beef, and salmon) and as carotenoids (provitamin A) from plant-based foods (such as spinach, mango, and carrots) [129]. Chemically, vitamin A1 (retinol) consists of a β-ionone ring coupled to an unsaturated isoprenoid chain ending with a hydroxyl group [130]. Due to its fat-soluble nature, it easily accumulates within the body, particularly in the liver and adipose tissue. However, deficiency can occur and is one of the most widespread causes of blindness due to corneal ulcerations and conjunctival keratinization [5]. Furthermore, the presence of vitamin A in the retina is essential for the formation of rhodopsin, a membrane protein found in rod cells that enables night vision. The synthesis of this molecule starts from vitamin A, which, in the presence of retinol dehydrogenase and the cofactor NAD, is oxidized from an alcohol group to an aldehyde. With the intervention of retinal isomerase, the double bond is isomerized from cis to trans, resulting in 11-cis-retinal, which then binds with opsin to form rhodopsin [131] (Figure 5).

Vitamin B12

Vitamin B12, or cobalamin, is an essential water-soluble micronutrient, with its main source of intake being animal proteins. It plays a key role in the synthesis of myelin, and its deficiency can lead to a multitude of pathologies, including peripheral neuropathy, ineffective erythropoiesis, subacute combined degeneration, megaloblastic anemia, and especially, optic nerve atrophy. It also has notable antioxidant activity thanks to the modulation of the NF-κB protein [5,132].

Vitamin C

**Figure 6:**
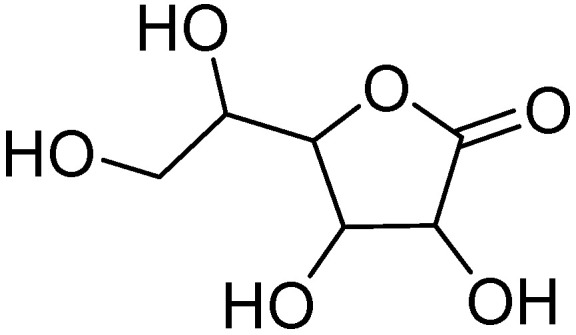
Structure of vitamin C.

Vitamin C or ascorbic acid (Figure 6) is a low-molecular-weight, water-soluble vitamin that cannot be synthesized by humans and must be obtained through the diet from foods such as citrus fruits, broccoli, and strawberries [133].

Structurally, vitamin C is characterized by a ring with an ethyl diol side chain and two hydroxyl groups that can act both as hydrogen bond donors and acceptors, unlike ketone and ether groups, which only act as hydrogen bond acceptors [134]. It plays a key role in the detoxification of ROS alongside vitamin E (tocopherol) and glutathione. Vitamin E is oxidized by donating a hydrogen atom to a radical species, becoming a “suicide molecule.” Ascorbic acid then intervenes by restoring tocopherol through its own oxidation to dehydroascorbic acid, which is later regenerated by glutathione. This mechanism does not occur under persistent oxidative damage, where dehydroascorbic acid undergoes irreversible degradation. Furthermore, at high doses, vitamin C can act as a pro-oxidant, increasing oxidative damage [135].

Vitamin C is found in high concentrations in the eye’s aqueous and vitreous humor, at levels 20–70 times higher than in the plasma [136]. Inside the eyeball, it helps prevent the penetration of UV rays by absorbing them and contributes to the elimination of radical species such as superoxide radicals, hydrogen peroxide, reactive nitric oxide, and hydroxyl radicals [137]. Under physiological conditions, vitamin C exhibits antioxidant, immunomodulatory, antithrombotic, antiviral, and wound-healing properties, and prevents lipid peroxidation of membranes [138]. Studies have shown that individuals deficient in vitamin C are more susceptible to developing cataracts, with a clear correlation between this vitamin and lens health [139].

Consequently, in 2000, the Institute of Medicine of the United States proposed a Recommended Daily Allowance of 90 mg/day for men and 75 mg/day for women [140]. However, more recent research suggests that this dose is too low, and that the daily requirement, regardless of gender, should be around 200 mg/day to positively influence ocular health [141]. Further studies have demonstrated that, in patients with diabetes, supplementation with vitamins C and E increases tear secretion and enhances the stability of goblet cells, leading to a decrease in the production of nitric oxide (NO). This is significant because NO contributes to the formation of reactive nitrogen species such as peroxynitrite, an oxidant that causes ocular inflammation [142].

Studies have shown that higher dietary intake of vitamin C is associated with a lower risk of cataract development. Vitamin C, as part of the antioxidant formulation used in AREDS-1 and AREDS-2, has been shown to reduce the risk of progression to advanced AMD when combined with other antioxidants and zinc. Vitamin C may help protect ocular tissues by scavenging reactive oxygen species and improving vascular health in the optic nerve head. Although evidence is less robust than for cataracts or AMD, maintaining adequate vitamin C levels may provide adjunctive support in glaucoma management [143,144,145].

##### Minerals

Like vitamins, many minerals that function as important cofactors in various enzymatic reactions also play a powerful antioxidant role within the human body. Among the most notable is selenium, which is present both in inorganic forms, such as selenites and selenates, and in organic forms, such as seleno-amino acids (in plants, the most common are seleno-methionine and methyl-selenocysteine), seleno-peptides, and seleno-proteins found in various foods such as meat, eggs, cereals, and seafood. Studies have shown that some seleno-proteins, including glutathione peroxidase and thioredoxin reductase, act as intracellular antioxidants, preventing oxidative damage in various ocular diseases [146,147]. Similarly, supplementation with this mineral provides benefits in various pathologies such as atherosclerosis, hypercholesterolemia, phenylketonuria, and type 1 and 2 diabetes mellitus. Another key mineral is zinc, an essential trace element fundamental for the activity of over 2800 macromolecules and more than 300 enzymes, as it is indispensable for proliferation, differentiation, apoptosis, and cellular communication. Zinc is found in high concentrations within the retina and choroid of the eye, where it interacts with vitamin A for the synthesis of rhodopsin [148,149]. Furthermore, it plays a significant role in immunomodulation, neurotransmission, and antioxidant defense. Zinc deficiency can lead to blindness (as a collagenase cofactor, its deficiency may cause corneal ulcers), anorexia, testicular atrophy, alopecia, epidermal hyperkeratinization, and delayed sexual maturation. Like zinc, copper is also found in significant quantities inside the eyeball, where it acts as a cofactor for various enzymes, such as superoxide dismutase and mitochondrial oxidative enzymes, which are essential for reducing oxidative damage caused by ROS.

#### 3.2.4. Phytochemicals

This class of nutraceuticals is characterized by the presence of chemical compounds of plant origin with specific roles in metabolic, physiological, and immunological processes. They occur naturally in a wide range, and many have demonstrated significant effects on ocular, neurodegenerative, and psychiatric diseases. The main classes used for ocular diseases are polyphenols (flavonoids and non-flavonoids), carotenoids, and quinones.

##### Polyphenols

This class of compounds is mainly divided into two types: flavonoid and non-flavonoid polyphenols. Flavonoids are characterized by the presence of three rings: a central pyran ring (called ring C) connected to two phenolic rings (called A and B). Their modification gives rise to numerous subgroups: flavones, chalcones, isoflavonoids, flavanones, isoflavonoids, flavonols, flavan-3-ols and anthocyanins [150]. These compounds can be found in many foods, including vegetables, berries, legumes, and fruits. [83]. The benefits of these metabolites are numerous, with the main ones being powerful antioxidant action throughout the body; the prevention of various forms of cancer, such as prostate and breast cancer; the regulation of diabetes mellitus; the exertion of antidepressant effects by acting on various neurotransmitter receptors and reducing levels of serotonin, noradrenaline, 5-hydroxyindoleacetic acid (5-HIAA), and dopamine; action on neuroinflammation secondary to ischemia–reperfusion injuries or neurodegenerative diseases; the regulation of transcription factors, signaling pathways, and gene expression; and reductions in neurotoxic mediators such as PGE2 [83,151,152,153]. Non-flavonoid polyphenols represent a diverse class of compounds structurally defined by a core aromatic ring substituted with one or more hydroxyl groups. The major subgroups comprise phenolic acids—subdivided into hydroxybenzoic and hydroxycinnamic acids—along with stilbenoids and lignans [154]. These molecules are widely distributed in vegetables, fruits, berries, and turmeric roots, and, like flavonoids, they exhibit a broad spectrum of beneficial biological activities [83]. A particular study, by Verma et al., shows that gallic acid has an inhibitory action on carcinogenesis due to its intervention in pathways such as the activation of ATM kinase, and the induction of intrinsic (cytochrome c) and extrinsic (Fas/FasL) apoptosis [155]. Moreover, studies by Liu et al. have established that syringic acid contributes to cardiovascular health after myocardial ischemia by reducing reperfusion damage through the activation of the PI3K/Akt/GSK-3 pathway, leading to a decrease in infarct size, mitochondrial apoptosis, and levels of CK-MB and LDH [156].

Curcumin

**Figure 7:**
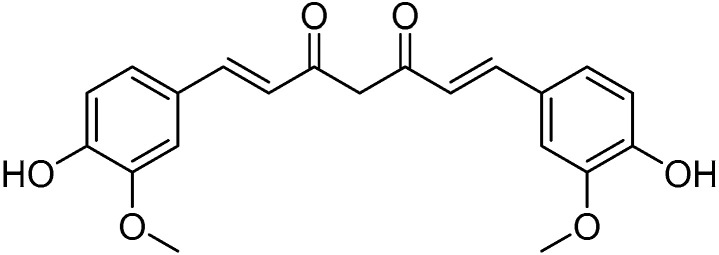
Structure of curcumin.

Curcumin (Figure 7) is a natural yellow–orange pigment belonging to the curcuminoid class and extracted from the rhizome of *Curcuma longa* (Linnaeus, 1753) (Zingiberaceae). This rhizome yields a powder rich in bioactive compounds, primarily curcumin (2 to 5%), along with its demethoxylated derivatives (demethoxycurcumin and bisdemethoxycurcumin), β-carotene, lycopene, epigallocatechin gallate, quercetin, and many others [157]. Often referred to as the “Golden Multitarget Nutraceutical,” curcumin has demonstrated a wide range of beneficial effects, including anti-inflammatory activity, anticancer potential, and efficacy in treating infectious diseases [158,159,160,161,162].

This molecule exerts its antioxidant effect by eliminating and inhibiting the production of various free radicals such as ROS and RNS. This function is primarily achieved through the inhibition of lipoxygenase/cyclooxygenase and xanthine dehydrogenase/oxidase. Furthermore, it modulates key antioxidant enzymes such as glutathione peroxidase (GSH-Px), catalase (CAT), and superoxide dismutase (SOD) [163,164]. It also has anti-inflammatory actions by activating the peroxisome proliferator-activated receptor (PPAR-γ) and reducing the expression of the IκBα gene, cyclooxygenase-2 (COX-2), PGE-2, interleukins 1, 6, and 8 (IL-1, IL-6, IL-8), and tumor necrosis factor (TNF-α) [165]. Due to its ability to counteract ROS, this molecule shows potential for therapeutic use in various angiogenesis-related disorders, particularly ocular conditions [166].

In studies by Munia et al., it was demonstrated that this molecule protects human retinal epithelial cells from cell death by decreasing the concentration of ROS inside the retina [167]. By inhibiting the pro-inflammatory interleukins IL-6, IL-8, and IL-1 within the conjunctiva, Li et al. demonstrated how this nutraceutical can be used to treat dry eye syndrome thanks to its anti-inflammatory effect [168]. Additionally, Lal et al., in their study on remedies for uveitis (inflammation of the uvea), demonstrated that patients suffering from chronic uveitis improved following oral curcumin supplementation via capsules (average dose of 75 mg/capsule) [169].

The main issue regarding the intake of this nutraceutical lies in its poor oral solubility and reduced bioavailability; however, exogenous administration for about eight weeks has been shown to effectively exert all its biological functions [170].

Quercetin

**Figure 8:**
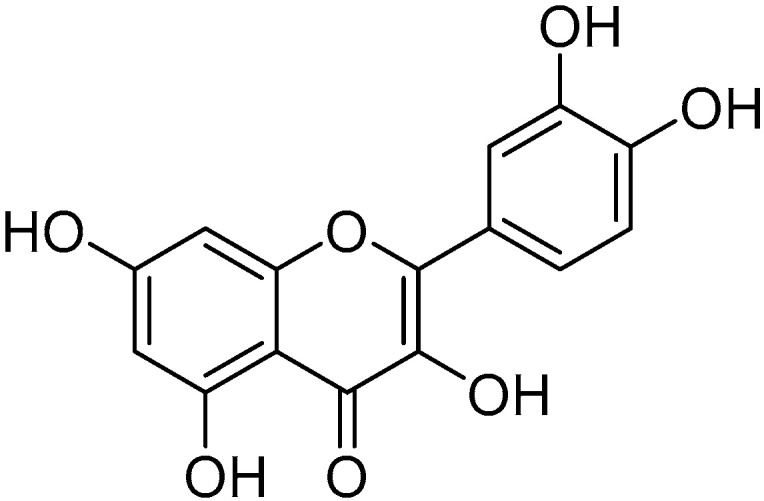
Structure of quercetin.

Quercetin (Figure 8) is a flavonol widely distributed in various types of fruits and vegetables (such as onions, capers, grapes and apples) possessing various beneficial effects including antioxidant, anti-inflammatory, anti-tumor, anti-aging, and immunomodulatory and metabolic disease-supporting properties [171].

Several studies led by Sanderson et al. have demonstrated that this molecule can protect the eye from cataracts induced by hydrogen peroxide and from retinal lesions caused by diabetes [172]. On the other hand, studies by Ola et al. suggest that quercetin may protect neurons in diabetic rats from damage caused by diabetic retinopathy by inhibiting caspase-3, improving levels of neurotrophic factors, and preventing neuronal apoptosis by increasing the amount of the anti-apoptotic protein Bcl-2 [173]. Wang et al. have demonstrated that quercetin protects the retina from oxidative stress and inflammation caused by exposure to light, suggesting its potential use in preventing the onset and progression of age-related macular degeneration [174]. Experimental use of quercetin in artificial tears, both individually and in combination with resveratrol, indicates that its topical application could represent a promising strategy for treating dry eye disease [175].

Resveratrol

**Figure 9:**
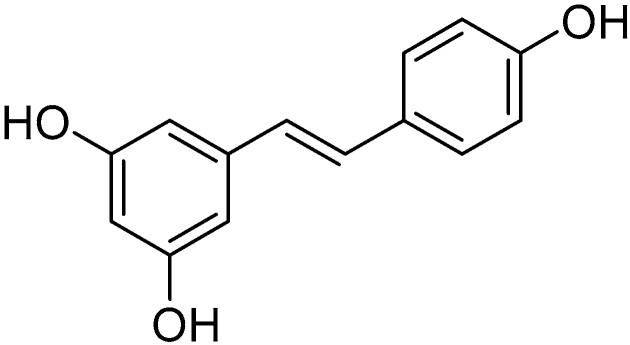
Structure of resveratrol.

Resveratrol (Figure 9) is a phytoalexin belonging to the stilbenoid class, mainly found in the seeds and skin of grapes. It possesses significant antioxidant, anti-inflammatory, and chemopreventive properties.

According to a study conducted by Doganay et al. on laboratory rats, resveratrol can effectively counteract the formation of cataracts induced by exposure to sodium selenite while increasing the levels of reduced glutathione in the crystalline lens, helping to limit the structural damage caused by hydrogen peroxide and inhibiting p53-mediated apoptosis [176]. Thanks to its anti-apoptotic effects, Luna et al. demonstrated that its intake is associated with a reduction in the expression of glaucoma markers caused by chronic oxidative stress in trabecular meshwork cells [177]. At the retinal level, resveratrol is also able to counteract damage caused by diabetes, apoptosis, the synthesis of pro-inflammatory cytokines, and the activation of the transcription factor NF-κB [178]. Finally, according to a study by Wattanapenpaiboon et al., its intake at concentrations of 50 and 100 mmol/L was found to inhibit the growth of retinal pigmented epithelium cells in vitro by 10% and 25%, respectively [179].

Epigallocatechin gallate (EGCG)

**Figure 10:**
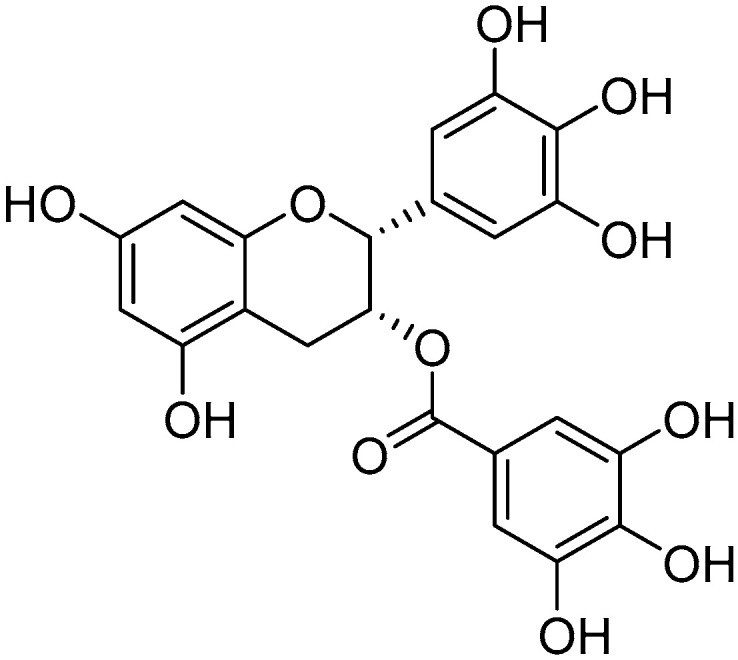
Structure of epigallocatechin gallate.

Epigallocatechin gallate (Figure 10) is the main flavonoid found in green tea, the polyphenols of which have remarkable antioxidant properties due to their ability to inhibit enzymes that generate reactive oxygen species (ROS).

In the study by Wung et al., it was observed that this molecule induces the enzyme HO^−1^ in endothelial cells through the activation of Akt and Nrf2, thus protecting these cells from oxidative stress caused by hydrogen peroxide [180]. Green tea extract has also been associated with the attenuation of cataracts and the treatment of glaucoma by protecting retinal neurons from damage caused by high intraocular pressure [181]. Moreover, it has been shown to inhibit vascular endothelial growth factor (VEGF) and the signaling pathways of extracellular signal-regulated kinase (ERK) 1/2 [182]. In diabetic rats, EGCG supplementation has been observed to increase GSH and SOD levels and reduce VEGF and TNF-α levels, suggesting its potential use in diabetic retinopathy [183]. In rabbits affected by dry eye syndrome, topical supplementation with EGCG and hyaluronic acid has demonstrated anti-inflammatory and mucoadhesive properties [184].

##### Carotenoids

**Figure 11:**
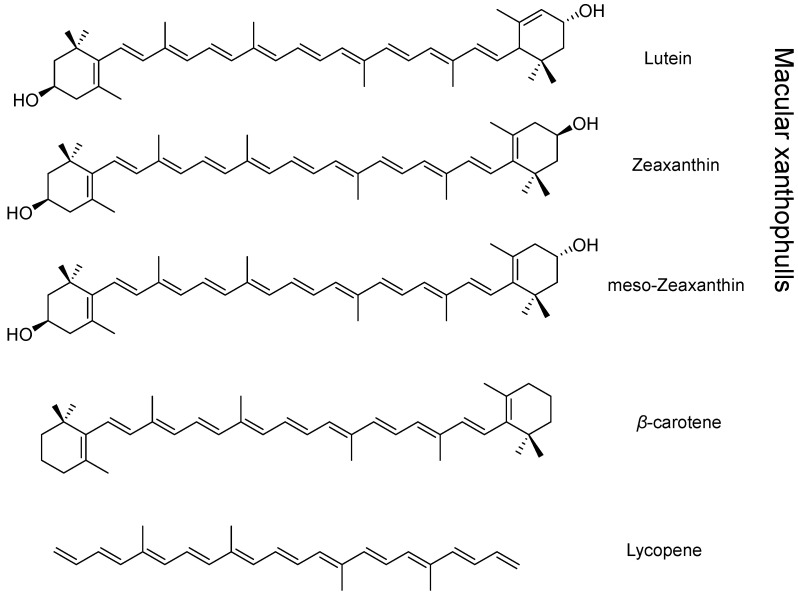
Structural difference between carotenes and xanthophylls.

Carotenoids are a group of fat-soluble pigments comprising over 600 natural compounds. They are found in a wide range of organisms, from plants to bacteria, but only about forty are regularly consumed through diet [185]. Chemically, they have a structure containing 40 carbon atoms arranged in eight isoprene units, which constitute the chromophore system responsible for their color. These compounds can be further subdivided into carotenes and xanthophylls (Figure 11).

The main function attributed to all carotenoids is their antioxidant activity; in addition, carotenes have pro-vitamin A activity [186]. Among the carotenes, the most important is β-carotene, which is present in most fruits and vegetables that tend to be orange or red in color. It has powerful antioxidant properties and helps prevent breast, esophageal, and bladder cancer. Furthermore, it has been noted that in laboratory rats, β-carotene can reduce the damage caused by thioacetamide by decreasing ACE and increasing SOD, CAT, and GSH levels [187,188]. Like β-carotene, lycopene has shown beneficial effects in the prevention of prostate cancer, in lowering blood pressure, and in reducing LDL cholesterol [189].

In the macula, the xanthophylls lutein, zeaxanthin, and meso-zeaxanthin predominate and are responsible for the color of the fovea; their intake, mainly through diet, significantly reduces the risk of developing age-related macular degeneration and cataracts [190]. While lutein and zeaxanthin must be obtained exclusively through a diet rich in vegetables and fruits pigmented mainly yellow and green, meso-zeaxanthin can be produced by the conversion of lutein within the eye [191]. Like carotenes, these compounds have strong antioxidant activity, mainly acting within the macula to neutralize oxidative reactions in photoreceptor cells. Additionally, they help reduce the formation of lipofuscin, which accumulates with age in the retinal pigment epithelium, where it can trigger cell apoptosis [192].

Carotenoids such as lutein and zeaxanthin accumulate in the lens and can neutralize reactive oxygen species, protecting lens proteins from oxidative stress. Epidemiological studies suggest that higher dietary intake of these carotenoids is associated with a reduced risk of cataract formation [193,194].

AMD is strongly linked to oxidative stress in the retina. Lutein and zeaxanthin, concentrated in the macula, filter blue light and act as antioxidants, protecting retinal cells. The AREDS2 trial demonstrated that supplementation with lutein and zeaxanthin reduces the risk of progression to advanced AMD, supporting their role in macular health [195,196].

Carotenoids, particularly lutein, may provide neuroprotection by reducing oxidative damage and maintaining retinal structure. Although clinical evidence is limited, preclinical studies suggest a beneficial effect on retinal ganglion cell survival [197,198].

##### Quinones

Quinones are a class of organic molecules derived from aromatic compounds, with a basic structure consisting of a conjugated cyclic diketone. These compounds are obtained from plants belonging to the Rubiaceae, Rhamnaceae, and Fabaceae families. The main representatives are anthraquinones (which can be further divided into monomers or dimers based on the structure of the nucleus), such as emodin, cascarine, chrysophanol, catenarin, and rhein. These compounds possess a variety of biological activities, including anti-inflammatory effects (by inhibiting TNF-α and IL-6) and antioxidant properties (by inhibiting lipid peroxidation and scavenging free radicals), as well as antimicrobial, anticancer, and laxative activities [191].

Coenzyme Q10

**Figure 12:**
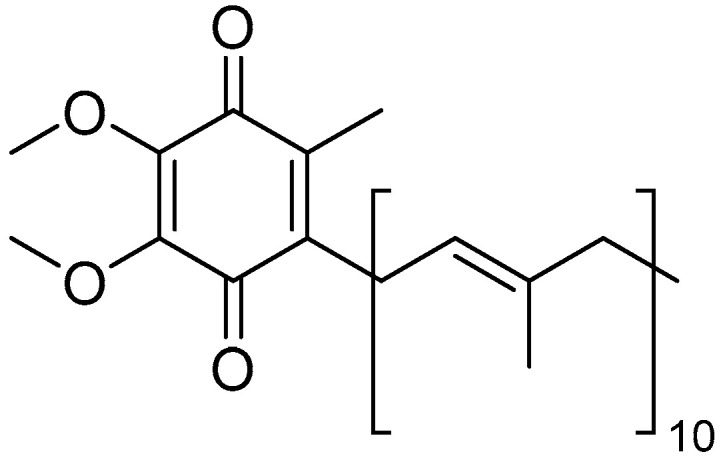
Structure of coenzyme Q10.

Coenzyme Q10 (CoQ10) (Figure 12), also known as ubiquinone, is a vital molecule found throughout the body, particularly in the mitochondria, where it plays a crucial role in cellular energy production.

CoQ10 is a cofactor for many mitochondrial enzymes responsible for ATP production. It transports electrons from complex I (NADH ubiquinone oxidoreductase) and complex II (succinate ubiquinone reductase) to complex III (ubiquinone cytochrome c reductase) [199]. When reduced, CoQ10 becomes a powerful antioxidant by decreasing the accumulation of ROS through direct action, as well as by regenerating the active form of vitamin E through indirect action [185]. Since the retina is the most metabolically active tissue in the body, with the highest energy consumption relative to its size, patients with CoQ10 deficiency may be more prone to developing retinopathies [200]. A study by Que et al. found that the retinas of young individuals (30 years old) contain higher concentrations of CoQ10 compared to those of elderly individuals (80 years old), highlighting the role of ROS in the aging process and in the development of conditions such as cataracts, age-related macular degeneration, glaucoma, atherosclerosis, and Alzheimer’s disease [201]. Likewise, experimental studies have shown that intravitreal administration of CoQ10 reduces apoptosis in retinal ganglion cells (RGCs) in cases of glaucoma [202].

#### 3.2.5. Herbs

Herbs are classified as nutraceuticals based on their chemical compounds, which possess various properties such as anti-inflammatory, antipyretic, diuretic, and analgesic effects. Among the most important herbs is *Vaccinium erythrocarpum* (Michx, 1803), which is rich in proanthocyanidin antioxidants capable of performing numerous functions, including the following: preventing urinary tract infections [203]; providing cardioprotective effects by maintaining healthy blood pressure, lipoprotein levels, and homocysteine levels; interfering with specific inflammation and oxidative stress pathways, such as NF-κB and Nrf2 [204]; and playing a key role in tumorigenesis by exhibiting direct cytotoxicity and inhibiting enzymes involved in cell proliferation [205]. Proanthocyanidins hold significant implications for ophthalmic health due to their potent antioxidant properties, and mitigate oxidative stress, a key pathogenic factor in a range of ocular disorders including AMD, DR, and cataract formation. By modulating redox-sensitive signaling pathways such as NF-κB and Nrf2, proanthocyanidins can attenuate chronic retinal inflammation, reduce vascular dysfunction, and preserve the integrity of retinal pigment epithelial cells. Additionally, their capacity to improve systemic vascular health—through the regulation of blood pressure, lipoproteins, and homocysteine—translates into enhanced ocular perfusion and a reduced risk of ischemic retinal injury. The anti-inflammatory and cytoprotective effects further contribute to maintaining corneal and retinal homeostasis, while their antitumorigenic activity suggests a potential role in reducing the risk of ocular surface neoplasia [203,204,205].

*Salix nigra* (Marshall, 1785) and *Lavandula angustifolia* (Mill., 1768) also have significant effects, demonstrating substantial anti-inflammatory and antioxidant activity through the suppression of pro-inflammatory cytokines, lipid peroxidation, and the accumulation of free radicals [206]. Several analyses by Aboutaleb et al. have shown that lavender helps restore antioxidant enzymes such as SOD, CAT, and GSH [207].

Lastly, *Ginkgo biloba*, which contains numerous flavonoid glycosides, is of considerable interest for ophthalmic applications and is being examined for its potential to prevent mitochondrial oxidative stress in glaucoma. It has been shown to inhibit the increase in intraocular pressure in laboratory rabbits and to prevent the adverse effects of dexamethasone on trabecular meshwork cells, leading to improvements in the deteriorated visual field of patients with open-angle glaucoma [208]. Additionally, *Ginkgo biloba* appears to delay the progression of age-related macular degeneration by preventing free radical damage to cell membranes [209]. Moreover, through the terpenoid ginkgolide B, it protects retinal ganglion cells by inducing an anti-apoptotic mechanism [210].

## 4. Comparative Discussion, Perspectives and Conclusions

Nutraceuticals represent a promising complementary strategy for the treatment and prevention of ocular diseases due to their antioxidant, anti-inflammatory, and neuroprotective properties. When we step back and compare the different categories of nutraceuticals in eye health, some clear patterns emerge.

Enzymes, such as pectinases and cellulases, show interesting biological activity in preclinical models, but their direct relevance to ophthalmology is still limited. At this stage, the evidence is modest, and translation will require better formulation and delivery strategies.

Probiotics, prebiotics, and symbiotics are a bit further along. While most of the strongest data come from gastrointestinal and immune research, we are now seeing emerging connections to ocular health, particularly through the so-called gut–eye axis. Small clinical studies and animal models hint at benefits for dry eye and even lens elasticity, and given their excellent safety record, probiotics hold good translational potential if we can design ocular-specific clinical trials.

Peptides occupy an interesting middle ground. Compounds like CAP37 demonstrate antimicrobial and wound-healing activity, and casein-derived peptides show vascular and pressure-lowering effects. The evidence is mainly preclinical, but the targeted mechanisms suggest real potential, especially if topical delivery can be optimized.

Carbohydrates such as fucoidan and laminarin are best known for their antioxidant and antithrombotic effects. Most of the work here is still in the laboratory, though the mechanisms point to possible roles in conditions like age-related macular degeneration or diabetic retinopathy. Their size and complexity, however, raise questions about absorption and bioavailability.

Moving to the fatty acids, omega-3 polyunsaturated fatty acids—EPA and DHA—stand out with stronger evidence. They have been studied extensively in cardiometabolic health and now increasingly in ophthalmology, where they influence intraocular pressure, retinal health, and inflammation. While results are mixed across studies, the mechanistic plausibility is strong, and these nutrients are safe, scalable, and clinically relevant.

Vitamins remain a cornerstone. Vitamin A and C have high-quality evidence supporting their importance in ocular health—whether through maintaining retinal function, protecting the lens, or strengthening antioxidant defenses. Vitamin B12 is somewhat less established but shows promise in neuroprotection. With vitamins, of course, dosing must be carefully managed to avoid toxicity [211,212,213].

Minerals such as zinc, copper, and selenium are similarly important. Zinc, for example, is critical for retinal function, and selenium contributes to antioxidant enzymes. The evidence is strong, but the therapeutic windows are narrow, meaning supplementation must be carefully tailored.

Polyphenols—including curcumin, quercetin, resveratrol, and EGCG—are widely studied for their anti-inflammatory and antioxidant properties. Most of the data are preclinical or early clinical, but the consistency of the mechanistic effects is striking. Their Achilles’ heel is poor bioavailability, though modern delivery systems like micelles and liposomes are helping overcome this limitation.

Carotenoids, especially lutein and zeaxanthin, are probably the most clinically validated of all nutraceuticals in ophthalmology. They are strongly linked to macular pigment density and protection against age-related macular degeneration. Here, both the evidence base and translational potential are very high.

Quinones, exemplified by coenzyme Q10, occupy a middle tier. They show clear mechanistic benefits in protecting retinal ganglion cells and supporting mitochondrial health, with some early human data. Bioavailability has been a hurdle, but improved formulations are moving this forward.

Finally, herbal medicines such as bilberry, willow bark, lavender, and ginkgo biloba show intriguing effects, particularly ginkgo in glaucoma. The main challenge here is consistency—herbal preparations vary greatly in active content, and safety must be monitored, especially for interactions with conventional drugs.

Therefore, if we rank them broadly, vitamins, minerals, carotenoids, and omega-3 fatty acids have the strongest and most immediate clinical relevance. Polyphenols, coenzyme Q10, and certain peptides look highly promising but depend heavily on formulation advances. Probiotics, carbohydrates, and herbs are emerging players, biologically plausible but in need of more robust ocular-specific clinical trials before they can enter mainstream ophthalmology.

Several studies have demonstrated the efficacy of specific nutrients—such as vitamins C and E, carotenoids (lutein and zeaxanthin), omega-3 fatty acids, and polyphenols—in slowing the progression of degenerative diseases including AMD, glaucoma, and diabetic retinopathy. However, despite these encouraging results, it is important to highlight that most of the evidence comes from observational studies or trials with methodological limitations. Therefore, further well-designed clinical trials with large sample sizes are essential to determine optimal dosages, long-term safety, and the true efficacy of nutraceuticals across various ocular conditions.

In conclusion, nutraceuticals may serve as a valuable adjunct therapeutic option, particularly within a preventive and integrative medicine framework, but they should not replace conventional therapies. Their use should always be considered within a personalized clinical context, taking into account the patient’s specific needs and the available scientific evidence.

## Figures and Tables

**Table 2:** The Age-Related Eye Disease Study, AREDS-1.

AREDS-1 Studies	
Vitamin C	500 mg
Vitamin E	400 UI
β-Carotene	15 mg
Zinc	80 mg
Copper	2 mg

**Table 3:** The Age-Related Eye Disease Study, AREDS-2.

AREDS-2 Studies	
Vitamin C	500 mg
Vitamin E	400 UI
Lutein	10 mg
Zeaxanthin	2 mg
Zinc	25 mg
Copper	2 mg
Omega 3	1 g (350 mg DHA and 650 EPA)

## Abbreviations

The following abbreviations are used in this manuscript:

BMI	Body Mass Index
Ile	Isoleucine
Pro	Proline
Val	Valine
RhLYZ	Recombinant lysozyme
DMS	Senile Macular Degeneration
DED	Dry Eye Disease
ROS	Reactive oxygen species
CNS	Central Nervous System
SAP	Standard Automated Perimetry
FDT	Frequency-Doubling Technology
SWAP	Short Wavelength Automated Perimetry
VEGF	Vascular Endothelial Growth Factor
OCT	Optical Coherence Tomography
OCT SLO	Scanning Laser Ophthalmoscopy OCT
AREDS	Age Related Eyes Disease Study
DR	Diabetic Retinophaty
DME	Diabetic Macular Edema
DHA	Docosahexaenoic acid
EPA	Eicosapentaenoic acid
PDR	Proliferative Diabetic Retinopathy
NPDR	Non-Proliferative Diabetic Retinopathy
SOD	Superoxide Dismutase
CAT	Catalase
GSH-Px	Glutathione Peroxidase
PUFA	Polyunsaturated Fatty Acids
EFSA	European Food Safety Authority
DPPH	2,2-diphenyl-1-picrylhydrazyl
IL-1	Interleukin 1
IL-5	Interleukin 5
IL-10	Interleukin 10
IL-12	Interleukin 12
IL-6	Interleukin 6
TNF-α	Tumor Necrosis Factor Alpha
FOS	Fructo-oligosaccharides
TOS	Galacto-oligosaccharides
GOS	Gluco-oligosaccharides
SOS	Soya-oligosaccharides
ACE	Angiotensin-converting enzyme
Ile-Pro-Pro	Isoleucine-Proline-Proline
Val-Pro-Pro	Valine-Proline-Proline
IL-7	Interleukin 7
IL-15	Interleukin 15
IFN-γ	Interferon Gamma
IL-1β	Interleukin 1*β*
AGE	Essential Fatty Acids
LA	Linoleic Acid
ALA	*α*-Linolenic Acid
AA	Arachidonic Acid
PGE2	Prostaglandin E2
IOP	Intraocular Pressure
RGC	Retinal Ganglion Cells
PEX	Pseudoexfoliative Glaucoma
ELOVL2	Elongation of Very-Long-Chain Fatty Acids-Like 2
VLC-PUFA	Very Long Chain PUFA
NF-kB	Nuclear Factor kappa-light-chain-enhancer of activated B cells
UV	Ultraviolet
IOM	Institute of Medicine
RDA	Recommended Daily Intake
NO	Nitric Oxide
ONOO^−^	Peroxynitrite
5-HIAA	5-Hydroxyindoleacetic acid
Fas/FasL	Fas and Fas Ligand
CK-MB	Creatine Kinase-MB
LDH	Lactate Dehydrogenase
RNS	Reactive Nitrogen Species
IκBα	Inhibitor of NF-κB Alpha
PPAR-γ	Peroxisome Proliferator-Activated Receptor Gamma
Bcl-2	B-cell lymphoma 2
EGCG	Epigallocatechin gallate
Akt	Protein Kinase B
Nrf2	Nuclear factor erythroid 2-related factor 2
ERK	Extracellular Signal-Regulated Kinase
ATP	Adenosine Triphosphate
CoQ10	Coenzyme Q10
